# The Effect of Heat Aging on the Microstructure and Properties of Spray-Deposited AlZnMgCu Alloy Extruded Plates

**DOI:** 10.3390/ma17153706

**Published:** 2024-07-26

**Authors:** Chen Chen, Di Feng, Zhiping He, Yichao Zhu, Zhiyuan Tao, Yinhui Xu, Haoran Wang, Jingtao Wang, Ying Liu

**Affiliations:** 1 China Helicopter Research and Development Institute, Jingdezhen 333001, China; hezp@avic.com (Z.H.);; 2 Department of Materials Science and Engineering, Jiangsu University of Science and Technology, Zhenjiang 212003, China; t18012365088@163.com (Z.T.); 18761047321@163.com (Y.X.); 18761047221@163.com (H.W.); 3 Department of Materials Science and Engineering, Nanjing University of Science and Technology, Nanjing 210094, China; jtwang@njust.edu.cn (J.W.); liuying517@njust.edu.cn (Y.L.)

**Keywords:** heat aging, heating rate, termination temperature, spray deposition, AlZnMgCu alloy, aging behavior

## Abstract

The heat-aging process, a practical aging technology that not only improves the comprehensive performance of Al alloys but also reflects the requirements of short processes, has an extremely practical significance. The effects of the heating rate and termination temperature on the “heat-aging” behavior of a spray-deposited AlZnMgCu alloy hot-extruded plate were investigated using hardness, electrical conductivity, room-temperature tensile strength, exfoliation corrosion experiments, and transmission electron microscopy microstructure (TEM) observation. The results show that as the termination temperature increases, the hardness of the spray-deposited AlZnMgCu alloy first increases to a peak and then rapidly decreases, while the electrical conductivity continues to increase. The increase in the heating rate improves the peak hardness corresponding to the termination temperature. The heat treatment process of heating at a speed of 20 °C/h to 200 °C after the spray deposition has similar mechanical and corrosion resistance properties to the RRA process and can effectively reduce the heating time from 40 h to 8 h, thus establishing a heat treatment process for spray-deposited AlZnMgCu alloy extruded plate with high aging efficiency.

## 1. Introduction

Higher mechanical and corrosion resistance of lightweight AlZnMgCu alloys is required in the modern aviation and space industries [1,2]. For the ultra-high-strength AlZnMgCu alloy, the macro-segregation degree and the hot cracking phenomenon in semi-continuous casting can be effectively reduced using spray deposition technology. The rapid solidification process in spray deposition not only increases the alloy element content but also refines the grains or the primary phases to finer and equiaxed states [3,4,5,6]. As the final heat treatment, aging is very important to improve the performance of the heat-treatable aluminum alloy. In order to fully unleash the potential of the fine structure and uniformity of compositions, it is crucial to explore the suitable aging technology for spray-deposited AlZnMgCu alloys.

At present, aging technology for spray-deposited AlZnMgCu alloys involves single-stage peak aging (SA), double-stage overaging, regression and re-aging (RRA), and the non-isothermal aging process (NRRA). It is well known that the aluminum alloy in its peak aging state conducted via a single heat preservation stage can obtain the highest hardness or strength but the poorest stress corrosion cracking (SCC) resistance. The overaging achieved via a double heat preservation stage, characterized by a low–high temperature evolution, can provide excellent SCC resistance but poor mechanical properties [7,8]. Research has indicated that regression, including a high temperature (about 190~200 °C) stage, can effectively disrupt the grain boundary precipitates (GBPs) while re-dissolving the intragranular precipitates (IGPs) smaller than the critical size [9,10,11]. Regression reduces the coarsening degree of IGPs when compared to double aging. Moreover, the finial mechanical properties can also be improved through re-aging. Therefore, the mechanical properties and corrosion resistance of high-strength aluminum alloys can be synergistically improved through the comprehensive regulation of IGPs and GBPs after regression and re-aging (RRA). However, RRA technology only theoretically confirmed the possibility of the synergistic improvement of the mechanical properties and corrosion resistance of AlZnMgCu alloys. It is almost impossible to achieve an industrial application due to the very narrow time window of regression [12]. For example, an optimized regression time is only several minutes when conducted at 200 °C. A severe coarsening of the IGPs will occur even with a slight extension of the regression time, causing a sharp decrease in the strength. Therefore, it is difficult to apply RRA to the heat treatment of large-scale components. According to the background of the national slogan of “energy conservation and emission reduction”, the development of a practical aging technology that not only improves the comprehensive performance of Al alloy but also reflects the requirements of short flow has extremely practical significance.

Large-scale aluminum alloy components inevitably experience heating or cooling processes during heat treatment, which inspires researchers to think about the rational utilization of the non-isothermal temperature field. Staley [13] first proposed a non-isothermal aging process and studied the microstructure and properties of the 7085 aluminum alloy under different heat-aging conditions. It was found that the mechanical properties of the 7085 aluminum alloy were excellent after heating at a rate of 13.9 °C/h for 10 h. Jiang et al. [14,15] found that during the cool-aging process, the continuous decrease in temperature inhibits the coarsening of precipitates. Instead, the secondary precipitation behavior is induced. In non-isothermal aging, the heating rate, the termination temperature (also defined as the highest temperature for heating—Ts), and the cooling rate are generally considered the most critical process parameters. An increase in conductivity but a decrease in hardness were obtained with an increase in Ts or a decrease in cooling rate. When the AlZnMgCu alloy is cooled at a rate of 10–20 °C/h within the range of 180~190 °C, an ideal match of strength and corrosion resistance can be achieved. Jiang [16] focused on studying the evolution of GBPs and precipitate-free zones (PFZs) during non-isothermal aging. The study showed that the GBPs maintained a continuous and a chain-like distribution feature during the low-temperature stage in heating, and no PFZ appeared. As the temperature increased, the GBPs began to coarsen and were separated. The PFZ significantly widened simultaneously. During the non-isothermal aging process, increasing the Ts or decreasing the cooling rate can promote the coarsening degree and the discontinuous distribution of GBPs, which is beneficial for improving the SCC resistance of the AlZnMgCu alloy.

Peng et al. [17] used a combination of enhanced solid solution (EST) and non-isothermal aging (NIA) methods to treat the rolled 7050 aluminum alloy. Their research showed that during the heat aging, the IGPs were mainly the solute atom segregation zones (GP zone) and the η′ phases. As the temperature increased, the precipitation driving force increased. The average size and the volume fraction of precipitates also rapidly increased. The main precipitates during the cooling stage were the η′ phase. Feng Di et al. [18] applied non-isothermal aging to RRA technology and referred to it as NRRA (non-isothermal “regression and re-aging”). By comparing and summarizing the precipitation behavior of GBPs in the 7055 aluminum alloy during the isothermal and non-isothermal RRA treatment, reference [19] established a non-isothermal regression dynamic model for GBPs. The model can effectively predict the size of GBPs at any time during the non-isothermal regression. Li et al. [20] performed single-stage aging (CA), double-stage aging (DA), and non-isothermal RRA (NRRA) treatments on a spray-deposited Al-8.15Zn-2.46Cu-1.97Mg-0.13Zr alloy. The order of strength levels under the different aging conditions is T6 > NRRA > T7, and the order of corrosion resistance is NRRA > T7 > T6. It is evident that the alloy treated with NRRA has excellent comprehensive properties.

As the simplest non-isothermal aging technology, heat aging [21,22,23] can be regarded as the accumulation of countless short-term isothermal aging stages. Every short-term isothermal aging stage has a different aging temperature. Therefore, the microstructure and property evolution of a spray-deposited AlZnMgCu alloy hot-extruded sheet during a non-isothermal heat-aging process were studied in the current study. The effects of the heating rate and termination temperature on the strength and SCC resistance were also analyzed and are discussed in detail. At the same time, the efficiency of thermo-treatment and the comprehensive properties of the AlZnMgCu alloy were compared between heat aging and single-stage peak aging (SA), double-stage overaging, and RRA. An optimized heat-aging technology prototype was ultimately established.

## 2. Experiment

The material used in this experiment is a spray-deposited AlZnMgCu alloy hot-extruded plate provided by Jiangsu Haoran Spray Deposition Co., Ltd. (Zhenjing, China). The chemical compositions (mass fraction, %) are Zn 8.12, Mg 2.04, Cu 2.38, Zr 0.12, Fe 0.041, Si 0.031, and residual Al. After the hot extrusion under 420 °C and 0.5 m/min with an extrusion ratio of 16, the two-stage solid solution (460 °C, 2 h + 480 °C, 2 h) was carried out in the high-temperature box resistance furnace. Room-temperature water quenching was adopted due to the severe quenching sensitivity of the AlZnMgCu alloy. The quenching transfer time was limited to about 5 s.

Three different heating rates (10 °C/h, 20 °C/h, and 40 °C/h) were employed for the immediate heat treatment of the AlZnMgCu alloy following quenching. The heating temperature ranged from the ambient temperature to 220 °C. After heating to the different termination temperatures (*T_s_*), the specimens were cooled immediately into room-temperature water to retain the aging state. In order to compare the aging efficiency and strengthening effect, this study simultaneously conducted single-stage aging (120 °C, 16 h-SA), double-stage aging (120 °C, 6 h + 160 °C, 24 h-DA), and RRA treatment (105 °C, 24 h + 190 °C, 10 min + 120 °C, 16 h) on spray-deposited AlZnMgCu alloy hot-extruded plates.

The trial samples underwent a series of time-dependent processes, and their hardness, electrical conductivity, tensile strength, resistance to pitting corrosion, and microstructural observation were systematically evaluated and analyzed in order to analyze the precipitation behavior and also to reveal the properties evolution rule.

Vickers hardness tests were conducted using an HV-10B hardness tester with a load of 1000 g for 15 s. The electrical conductivity (% IACS) was measured using an FD101 eddy current conductivity tester. The hardness and conductivity test results were taken as the average of three parallel specimens. According to GB/T228.1-2010 standard [24], a room-temperature tensile test was carried out on a UTM 5105X electronic universal testing machine for different aging-treated specimens. Three parallel specimens were conducted for every property test. The tensile direction was the extrusion direction and the tensile rate was 1 mm/min.

Exfoliation corrosion experiments were used to visually evaluate the corrosion resistance of aged materials under different precipitation states according to GB/T 22639-2022 standard [25]. The LTD-RD section of the specimen was exposed to an EXCO solution of 234 g/L of NaCl, 50 g/L of KNO_3_, and 6.3 mL/L of HNO_3_ at 25 ± 3 °C for a total of 48 h. The specimens were placed horizontally in the exfoliation corrosion solution. The corroded surface of the specimens should be more than 1 cm away from the liquid surface. The ratio of solution volume to the surface area was 20 mL/cm^2^. The 24 h- and 48 h-corroded specimen photographs were collected using a metallographic microscope and rated.

The precipitates in the grain interior and on the grain boundaries under different aging processes were observed via JEM-2100F transmission electron microscopy (TEM). The TEM specimen with 3 mm diameter and 0.08 mm thickness was twin-jet electropolished in a solution of nitric acid and methanol (3:7 in volume) at about −30 °C. The test acceleration voltage for TEM observation was 200 kV. High-resolution transmission electron microscopy (HRTEM) observations were also utilized to statistically analyze the size arrangement and the average size of precipitates.

## 3. Results

For the convenience of expression, H was used to represent the heat aging. Therefore, the H-termination temperature-heating rate represents the different heat-aging processes. For example, H-160-20 indicates that the specimen was heated from ambient temperature to 160 °C at a heating rate of 20 °C/h and then subjected to water-cooling treatment. Different termination aging temperatures were selected. The hardness and conductivity of the water-cooled specimens at the selected temperatures were measured.

### 3.1. The Evolution of Hardness at Different Heating Rates

**Figure 1:**
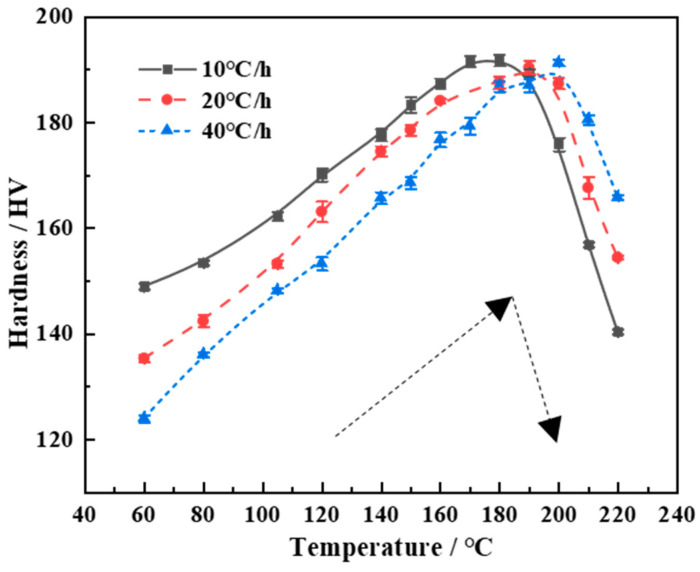
Hardness evolution curves of the spray-deposited AlZnMgCu alloy during heat aging.(Arrows represent the rise and fall of hardness by a sketch map pattern).

The hardness evolution curves at different heating rates are shown in Figure 1. The evolution rules are summarized as follows.

(1) At a fixed heating rate, the hardness first increases to its peak value and then decreases during the heat-aging process (Figure 1). This is consistent with the evolution rule of hardness during isothermal aging. Based on the correspondence between the hardness and aging temperature, it can be seen that in the high-temperature stage, after the peak hardness is obtained, the decrease rate (*V_C_*) of the hardness is significantly higher than the increase rate (*V_R_*) of the hardness before the peak hardness is obtained (compare the dashed arrow in Figure 1). For example, when the heating rate is 10 °C/h, the aging temperature corresponding to the peak hardness is about 180 °C. In the temperature range from 60 °C to 180 °C (Δ*T*_1_ = 120 °C), a hardness increment of approximately 40 HV can be obtained. The hardness increase rate V_R_ is about 3.3 HV/h. However, in the high-temperature range from 180 °C to 220 °C (Δ*T*_2_ = 40 °C), the hardness actually decreases by about 52 HV. The hardness decrease rate V_C_ is 13 HV/h, which is about 4 times more than V_R_. When the heating rate increases to 20 °C/h, the aging temperature corresponding to the peak hardness also increases to about 190 °C. In the temperature range from 60 °C to 190 °C (Δ*T*′_1_ = 130 °C), the hardness increment of approximately 55 HV is obtained. The hardness increase rate V_R_ is approximately 8.5 HV/h. In the high-temperature range from 190 °C to 220 °C (Δ*T*′_2_ = 30 °C), the hardness decreases by about 35 HV. The hardness reduction rate V_C_ reaches 23 HV/h. When the heating rate is 40 °C/h, the corresponding temperature for the peak hardness is about 200 °C. In the temperature range of 60 °C to 200 °C (Δ*T*″_1_ = 140 °C), the hardness increment is approximately 65 HV and V_R_ is about 18 HV/h. In the high-temperature range of 200 °C to 220 °C (Δ*T*″_2_ = 20 °C), the hardness only decreases by about 28 HV. However, the *V_C_* increases sharply to 65 HV/h, indicating a strong coarsening effect of precipitates.

From the comparison of hardness evolution rates, it can be seen that the hardening effect of the alloy slowly increases before the temperature corresponding to the peak hardness. After the peak hardening is reached, the softening effect of the alloy rapidly occurs. Therefore, it is necessary to choose a reasonable termination temperature for a given heating rate. That is to say, once the required SCC resistance level is reached, the heat aging should be interrupted as soon as possible to maintain the precipitation hardening effect.

(2) Although the heating rates are different, the evolution rule of hardness is the same. Moreover, the peak hardness levels do not differ significantly, ranging from 190 to 192 HV. However, the temperature corresponding to the hardness peak increases with the increase in heating rate. The hardening response rate increases with the heating rate increases. For example, specimens heated at 40 °C/h can achieve the same hardness in a relatively shorter aging time. As is well known, high-strength AlZnMgCu alloys are required not only to achieve good mechanical performance but also to possess both high strength and excellent corrosion resistance. Therefore, although rapid heating can accelerate the precipitation hardening rate, it may also lead to an insufficient aging time and unsatisfactory stress corrosion resistance. At the same time, it may also be unfavorable for practical operation due to the rapid evolution rate of hardness. More importantly, quick heating (such as 40 °C/h in the current research) requires matching higher termination temperatures, which is contradictory to “energy conservation and emission reduction”. Although the lower heating rate (such as at 10 °C/h in the current research) is relatively close to the temperature evolution rate in the large-scale aluminum alloy components, the lower aging efficiency also does not comply with the requirement of a short process characterized by “energy conservation and emission reduction”. Therefore, a moderate heat-aging rate (such as 20 °C/h) should be a more suitable heating parameter.

### 3.2. The Evolution of Electrical Conductivity at Different Heating Rates

**Figure 2:**
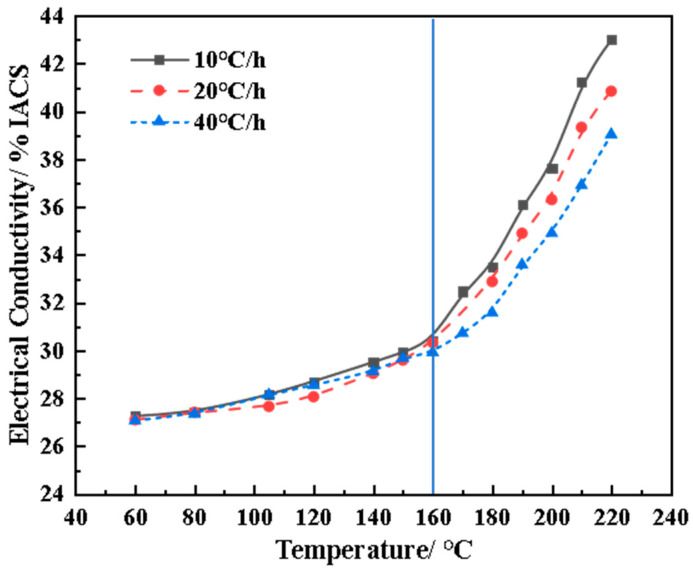
Conductivity curves of the spray-deposited AlZnMgCu alloy during heat aging. (The blue line divides the heating aging into two stages. The left stage below 160 °C shows a slow growth of conductivity.).

Figure 2 shows the electrical conductivity evolution curve under different heating rates. As shown in the figure, the conductivity continues to increase with the increase in the termination temperature *T*_s_, regardless of the heating rate. However, the increase in conductivity varies significantly across different temperature ranges. The conclusions can be summarized as follows: (1) When *T_s_* is below 160 °C, the increments of conductivity under the three heating rate conditions are all very small and almost the same, at about only 10%. (2) When the *T*_s_ exceeds 160 °C, the conductivity begins to rapidly increase with the increase in Ts. The higher the heat-aging rate, the lower the conductivity at the same termination temperature *T*_s_. This is evidently due to the lack of aging time. For extruded plates of the high-strength AlZnMg (Cu) alloy, the conductivity of 36% IACS is generally considered the standard representing good SCC resistance. It can be concluded that the optimal termination aging temperatures under three heating rate conditions are approximately 190 °C, 200 °C, and 210 °C, respectively.

Based on the three termination temperatures and the hardness evolution curve, the hardness values under three heating rate conditions can be obtained as 190 HV, 187.4 HV, and 186.3 HV, respectively. Although the hardness levels do not differ significantly, the specimens treated at 10 °C/h clearly have relatively high mechanical properties. Taking the ambient temperature (about 40 °C) as the starting temperature for aging, the shortest aging time is about 15 h when the effective heating rate is 10 °C/h. When the heating rates are 20 °C/h and 40 °C/h, the shortest aging times are 8 h and 4.3 h, respectively. Considering the heat-aging efficiency, it should be necessary to choose the treatment process with the shortest time. However, this process with a 40 °C/h heating rate requires a higher *T*_s_ (210 °C). As mentioned above, low energy consumption and emission reduction are the key requirements that are of great concern in practical production. Therefore, high-temperature aging may limit its industrial application. Heat aging at the rate of 10 °C/h results in an extension of the production cycle (15 h). Therefore, a heat-aging rate of 20 °C/h can not only meet the mechanical and corrosion resistance requirements but also effectively shorten the aging cycle. Moreover, Ts has also been reduced to a certain extent, which is completely in accord with the engineering requirements of short cycles and low energy consumption. Therefore, the subsequent analysis will be conducted using the specimens with a heating rate of 20 °C/h as the observation object.

### 3.3. The Exfoliation Corrosion Resistance at Different Heating Rates

**Figure 3:**
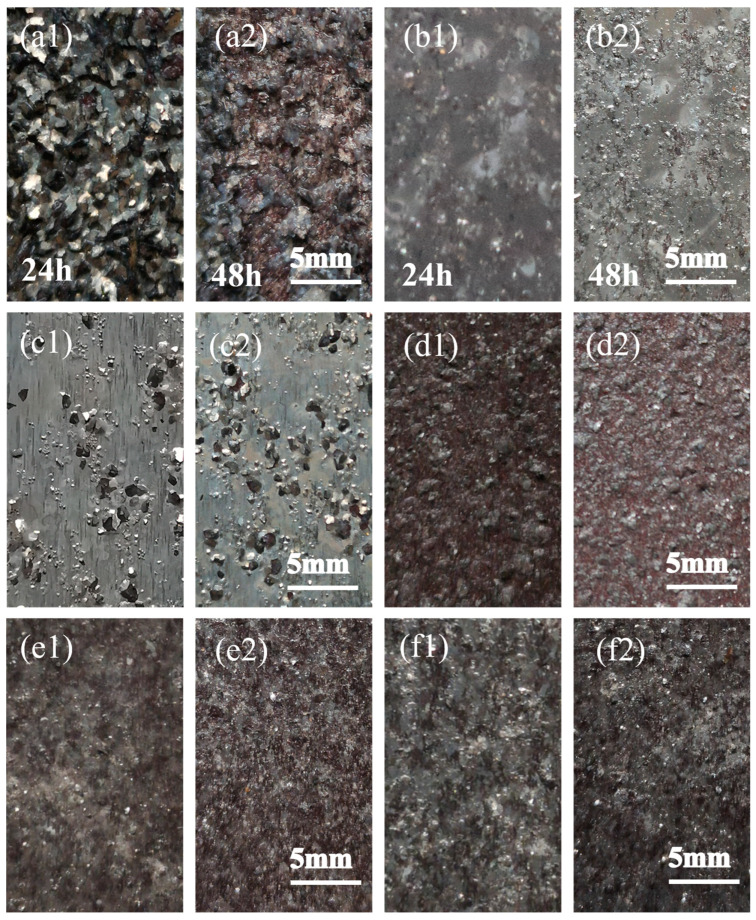
OM images of alloys after exfoliation etching for 24 h and 48 h, respectively, under different aging treatments: (**a1**,**a2**) single-stage aging; (**b1**,**b2**) double-stage aging; (**c1**,**c2**) RRA; (**d1**,**d2**) H-190-10; (**e1**,**e2**) H-200-20; and (**f1**,**f2**) H-210-40.

The exfoliation corrosion morphology of the spray-deposited AlZnMgCu alloy under different aging conditions is recorded in Figure 3. It can be seen in Figure 3a1,a2 that the severe stratification phenomenon appeared in the single-stage peak-aged specimen after 24 h of immersion corrosion. After 48 h of immersion, the color of the surface becomes dark red. The corrosion layer has peeled off. A large amount of black corrosion products are found at the bottom of the container, indicating that the corrosion has extended deeper into the alloy. According to the evaluation rule in the GB/T22639-2022 standard [25], the specimen under the T6 state shows the worst SCC resistance. The exfoliation corrosion resistance level can be assessed as ED.

After double-stage aging and RRA treatment, similar corrosion morphologies can be observed in both of the two specimens. There are almost no corrosion products at the bottom of the container. The specimen surface reveals a silver-gray color and no obvious delamination can be detected. Only a few corrosion pits appeared on the surface, which results in a tendency for exfoliation corrosion in local positions. Thus, only a slight peeling layer is formed. Evidently, double-stage aging and RRA can significantly improve the corrosion resistance of the spray-deposited AlZnMgCu alloy. The exfoliation corrosion resistance level can reach the EA level according to the GB/T22639-2022 standard [25].

The surface of the H-190-10 specimen shows a reddish-brown color. A slight layering occurs. Similar to the overaged specimen, such as the RRA-treated one, there is only small-scale peeling in local areas. The corrosion products are much fewer than those of the single-stage aging state while exceeding those of the double-stage aging and RRA specimens. The corrosion resistance of the H-190-10 specimen is evaluated as EB grade. The surface of the H-200-20 specimen shows a dark red color, which is similar to the state of the H-190-10 specimen. However, severe peeling and even bursting can be observed in local areas. The black corrosion products at the bottom of the container slightly increase. The corrosion cracking further expands into the matrix. Therefore, the exfoliation corrosion resistance level was reduced to the EB level; however, it still satisfied the performance criteria for the delamination corrosion of the AlZnMgCu alloy in AMS 4206B [26]. The surface of the H-210-40 specimen displays a severe peeling phenomenon. The corrosion products are only less than those in the single-stage-aging state. The corrosion gradually penetrated the interior. Thus, the exfoliation corrosion performance of the alloy in the H-210-40 state was further weakened, reaching the EC level.

### 3.4. The Tensile Strength at Different Heating Rates

**Figure 4:**
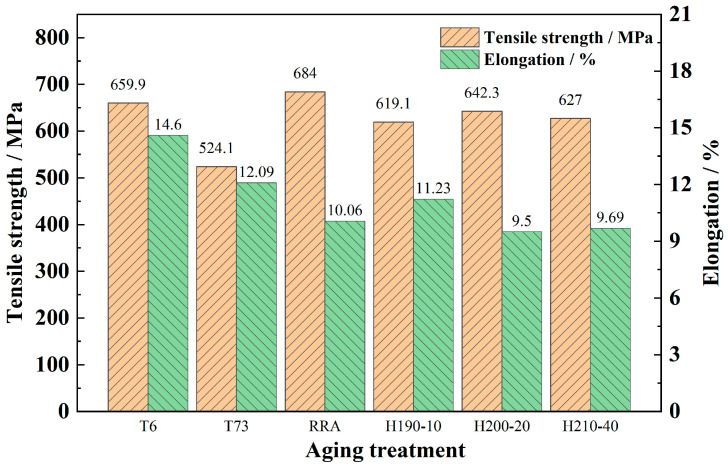
Mechanical properties of the spray-deposited AlZnMgCu alloy under different aging treatments.

The statistical diagram of the mechanical properties of the spray-deposited AlZnMgCu alloy after a two-stage solid solution and different aging treatments is shown in Figure 4. Similar mechanical properties with tensile strengths of 667 and 685 MPa are exhibited in the specimens under SA and RRA conditions, respectively. Under DA conditions, the elongation of the alloy is the highest (16.5%) but the strength decreases by about 20% (524.1 MPa). The ultimate tensile strength of the specimens heat-aged at different temperatures slightly decreased when compared to the SA and RRA conditions. Among them, the strength in the H-200-20 state is highest, which is about 642.3 MPa. Moreover, the elongation can also reach 11.3%, which demonstrates excellent mechanical properties. It should be pointed out that only 8 h were needed to obtain the excellent mechanical properties mentioned above. The efficiency of time hardening has significantly improved. The efficiency of aged strengthening has improved greatly via heat aging.

The comprehensive performance of the spray-deposited AlZnMgCu alloy under different aging processes is compared, and the results are shown in Table 1. From the table, it can be seen that after SA treatment, the strength and elongation of the alloy are higher than that of DA or heat-aging specimens, but the exfoliation corrosion performance is the worst. The anti-exfoliation corrosion level of the alloy under DA can reach EA, but the strength at this state is only 524.1 MPa. RRA enables the alloy to achieve both a high strength and good anti-exfoliation corrosion resistance level of EA. However, the whole aging process of RRA takes about an astonishing 40 h. Moreover, the regression time is so short that it makes it very difficult to achieve industrial applications. Fortunately, the heat-aging process of raising the temperature at a rate of 20 °C/h to 200 °C can achieve excellent comprehensive properties of the alloy in a relatively shorter period. It is particularly important to point out that, after H-200-20 was treated, the strength, the elongation, and the anti-exfoliation corrosion grade of the spray-deposited AlZnMgCu alloy reached 642.3 MPa, 11.3%, and EB, respectively, demonstrating excellent comprehensive properties. Additionally, the heat-aging process is easy to operate, and the time consumption is relatively shorter.

### 3.5. The Microstructure Observations of Heat Aging at 20 °C/h

#### 3.5.1. Intragranular Precipitates

**Figure 5:**
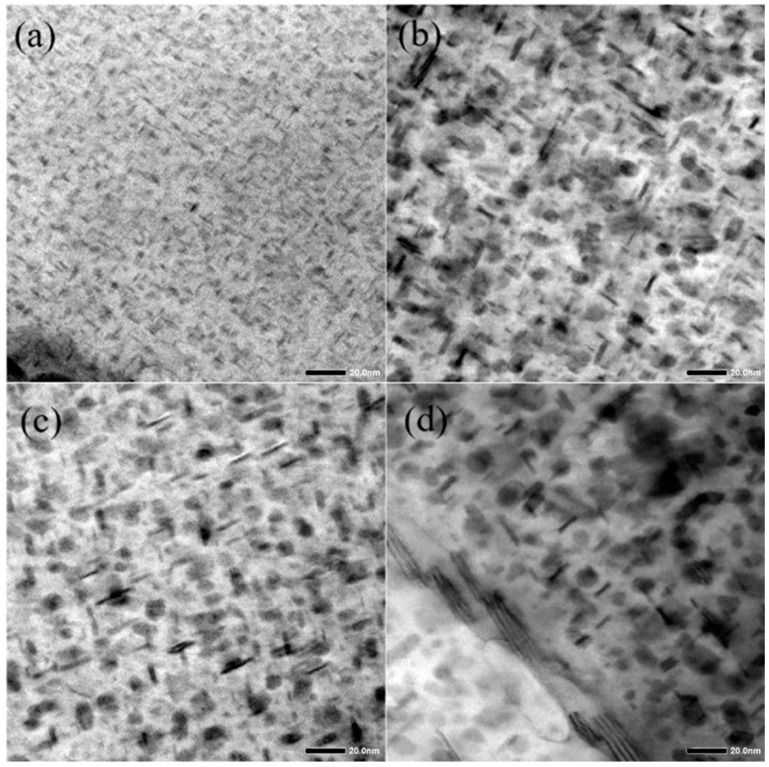
TEM images of precipitates of the spray-deposited AlZnMgCu alloy under different termination temperatures (heating rate of 20 °C/h): (**a**) 160 °C; (**b**) 190 °C; (**c**) 200 °C; (**d**) 210 °C.

The morphologies of intergranular precipitates (IPS) of the spray-deposited AlZnMgCu alloy during the heat-aging process with a 20 °C/h rate are shown in Figure 5. It can be seen that a large number of fine and dispersed nano-precipitates can be observed in the image with low resolution (Figure 5a) when Ts is 160 °C. The short rod-like and fine-needle-like nano-phases can be observed in the [100]Al projection, respectively. It can be preliminarily determined that these types of precipitates should be the fine η′ precipitates that keep a semi-coherent relationship to the Al matrix. The statistical data show that the size of η′ precipitates ranges from 3 to 5 nm when taking the size of the long axis into account, with an average size of approximately 3.9 nm.

When the T_s_ increases to 190 °C, the aging temperature already belongs to the range in which a retrogression behavior may occur [27]. A significant decrease in the volume fraction of the IPS can be seen in Figure 5b. Additionally, the size of the precipitate also significantly increases. It is surprising to observe that the fine precipitates with a size range of 1~2 nm distribute diffusely in the gaps of the larger-sized ones. Based on the investigation of the regression behavior, it can be inferred that during the process of heating to 190 °C, the small-sized phases precipitated in the low-temperature aging stage re-dissolve while the ones larger than the critical size continue to grow or even coarsen. This re-dissolved behavior leads to the appearance of a reduced volume fraction under the low-magnification condition. Meanwhile, with the continuous heat aging, a new cycle of nucleation and growth behavior of the η′ precipitates will also continue at the same time. The complicated aging behavior under the termination temperature of 190 °C results in a wider dimension scope of precipitate. Statistical results show that the precipitate size mainly distributes in the range of 2~8 nm, and the average size is about 7.0 nm.

Instead, the volume fraction of precipitates increases once again with the continuous heating of the specimen to 200 °C (Figure 5c). However, the average size of precipitates also increases. Similarly, fine precipitates can also be observed between the large-sized precipitates. According to the statistical results, the precipitate size is mainly distributed between 4 and 10 nm, with an average size increasing to about 8.5 nm. After further increasing the termination temperature to 210 °C, a decrease in the volume fraction of precipitates occurs. In addition, fine precipitates around the coarsened η′ phase are rarely observed. The precipitate sizes mainly distribute between 5 and 13 nm. An average size of around 10.9 nm is obtained, which is nearly three times higher than that of the H-160-20 specimen. It should be pointed out that the aspect ratio of the intragranular precipitates decreases clearly and the thickness of the disc significantly increases, as highlighted by the dashed box in Figure 5d. As mentioned above, the hardness of the H-210-20 specimen decreases to 168 HV. It is indicated that there is a high volume fraction of η precipitates under the overaged state, which results in a significant decrease in mechanical properties.

Based on the analysis of the whole heat-aging process, the precipitation sequence of heat aging can be seen as the same as that of isothermal aging, namely supersaturated solid solution (SSSS) → GP zone → η′ phase → η phase. However, different from the precipitation behavior in isothermal aging, nucleation, growth, and the coarsening behavior with different thermally stable precipitates occur simultaneously during non-isothermal aging. More importantly, there is also regression behavior in the mid-temperature range of heat aging (160–200 °C, especially around 190 °C). Therefore, there is a significant overlap in the nucleation, dissolution, growth, and coarsening behavior of the precipitates during the heat-aging process.

#### 3.5.2. Grain Boundary Precipitates

**Figure 6:**
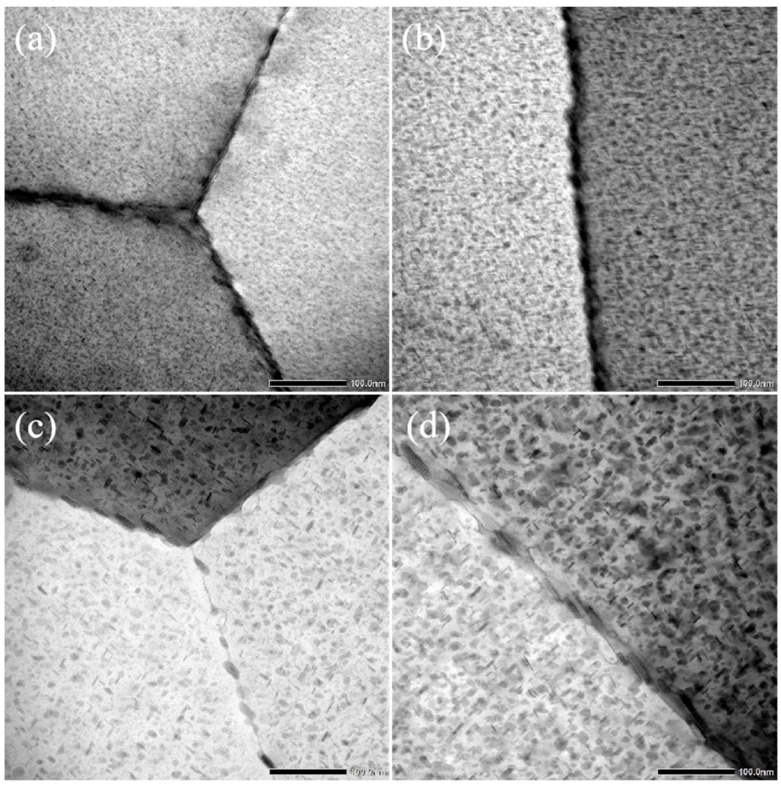
TEM images of the microstructure evolution in grain boundaries under different termination temperatures: (**a**) 160 °C; (**b**) 190 °C; (**c**) 200 °C; (**d**) 210 °C.

Figure 6 shows the grain boundary precipitate (GBP) morphologies of the spray-deposited AlZnMgCu alloy after aging with a heating rate of 20 °C/h. When the termination temperature is 160 °C, GBPs with a continuous distribution feature can be observed. In the H-160-20 specimen, the grain boundary is covered completely with the precipitates distributed in a strip-like shape. The discontinuities of GBPs are not evident, and no precipitate-free zone (PFZ) can be observed. When the termination temperature is raised to 190 °C, the coarse GBPs have been broken up into separated particles. The average interval of GBPs can be measured to be approximately 10.8 nm while the PFZ is still not evident.

When the termination temperature is 200 °C, the size of the long axis and short axis of the GBP both increase significantly. Conversely, the increase in precipitate size results in a decrease in the volume fraction of precipitates, thus achieving a complete disconnection feature of GBPs. At this overaged state, the average interval of GBPs is about 13.7 nm. A distinct and wide PFZ appears. The width of PFZ is approximately 25 nm. In further raising the termination temperature to 210 °C, the GBPs continue to coarsen. There is significant growth along the long-axis direction of precipitates. The degree of discontinuity is higher. The average interval of GBPs increases to about 22.6 nm, with a maximum value of nearly 50 nm. However, the broadening degree of PFZ has no significant change, only increasing to about 27.2 nm.

## 4. Discussion

### 4.1. The Effect of the Heating Rate on the Precipitation Behavior of Alloys

**Figure 7:**
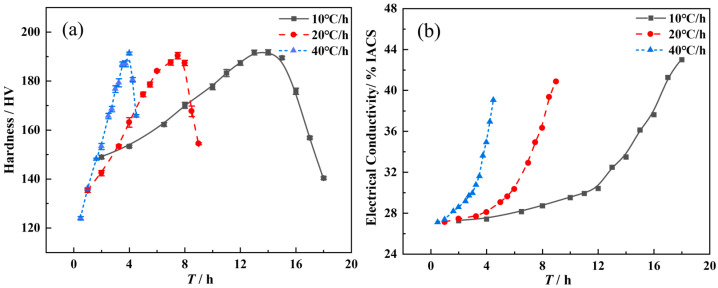
Effect of hardness–aging time curves (**a**) and electrical conductivity–aging curves (**b**) of the spray-deposited AlZnMgCu alloy under heat-aging treatments.

In non-isothermal aging, when the termination temperature is fixed, the heating rate determines the effective aging time. If the temperature rises too fast, the effective aging time will be shortened. Although slow heating can ensure sufficient aging time, the aging hardness rate decreases. Moreover, the specimen will also hold in the high temperature for a relatively longer duration, which not only leads to the dissolution of the pre-precipitates but also results in the growth and ever coarsening of the precipitates. When the termination temperature is not fixed, different heating rates provide varying degrees of “superheat” within the same duration. The “superheat” of the rapid heating is greater, and the aging hardening response is improved, and vice versa. However, under rapid heating, the growth and coarsening rate of the precipitate phase will also increase. Therefore, the heating rate is one of the decisive factors in heat-aging technology. The evolution curves of hardness and conductivity with aging time are shown in Figure 7a,b, respectively. It can be more clearly seen from the figure that during the age-hardening stage, rapid heating results in higher hardness and conductivity after the same aging time. Of course, the rapid softening phenomenon can also be observed.

### 4.2. The Effect of the Termination Temperature on the Precipitation Behavior

The typical microstructural parameters of IPs, GBPs, and PFZ of the spray-deposited AlZnMgCu alloy during the heat aging with a 20 °C/h rate are shown in Table 2.

**Figure 8:**
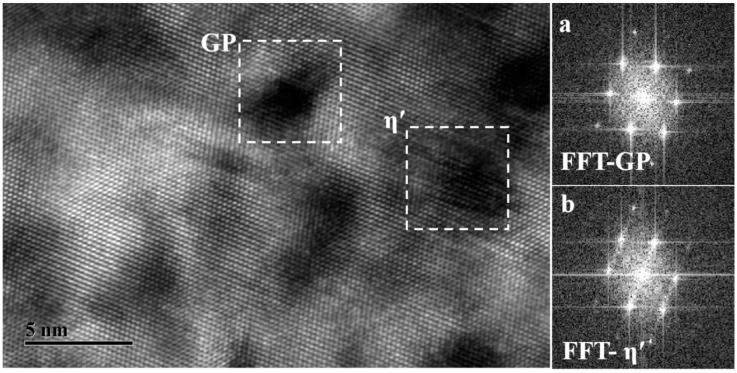
HRTEM image of the microstructure of the H-160-20 specimen: (**a**) FFT-GP; (**b**) FFT-η′.

According to the microstructural characteristics and properties evolution, it can be seen that nucleation and restricted growth are the main behaviors during the initial stage of heat aging, in which the volume fraction of precipitates is higher with smaller sizes. The high-resolution electron microscopy (HRTEM) images of the H-160-20 specimen are shown in Figure 8.

The black ball-like precipitates with an average size of 3 nm can be observed clearly in Figure 8. In addition, the plate-like precipitates with their bottom layer parallel to the {111}Al and extending along the <111>Al direction also exist. The results of the fast Fourier transform (FFT) are shown in a and b, respectively. The stress field contrast can be seen clearly in Figure 8a. The stripes along the <111>Al direction can be detected faintly, which are identified as fine GP zones or “clusters”. A multi-atomic layer structure can be seen in Figure 8b, showing that the typical morphology belongs to η′. As we all know, the GP zone/clusters and the non-equilibrium η′ exhibit coherent and semi-coherent relationships with the matrix, respectively, which results in a strong coherent strain-strengthening effect. Thus, the hardness of the H-160-20 specimen can be improved from 98 HV in the solid solution state to 184 HV, as is shown in Table 2. In other words, a certain supersaturation degree of solute atoms and vacancy are still retained due to the underaged state of H-160-20. The intense electron scattering resulting from the point defect leads to low conductivity.

**Figure 9:**
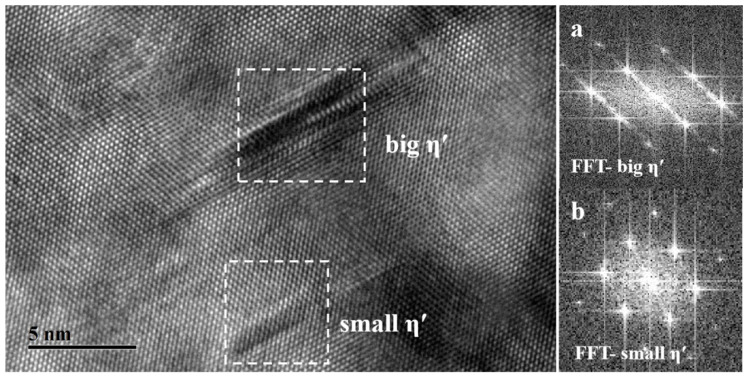
HRTEM image of the microstructure of the H-190-20 specimen: (**a**) FFT-GP; (**b**) FFT-η′.

Figure 9 shows a high-resolution electron microscopy (HRTEM) image of the H-190-20 sample, as well as the corresponding FFT of the precipitates. It can be seen from Table 2 that the hardness of the H-190-20 specimen has increased to approximately 190 HV, which corresponds to the peak aging state. Two types of multi-layer atomic structure precipitates with significant size differences can be observed. Considering the FFT analysis, it can be concluded that the precipitates under the H-190-20 state are mainly the metastable η′ precipitates. Further analysis indicates that the η′ with a larger size results from the growth of undissolved pre-precipitates, while the finer ones result from the growth of new nuclei. Corresponding to the increase in η′ in volume fraction, almost no GP zone and clusters can be observed, indicating an adequate decomposition of SSSS. The η′ maintains a semi-coherent relationship with the matrix. As the average size increases from 3.9 nm at H-160-20 to 7.2 nm, it is more effective in hindering the sliding of dislocations, resulting in a peak hardness level.

**Figure 10:**
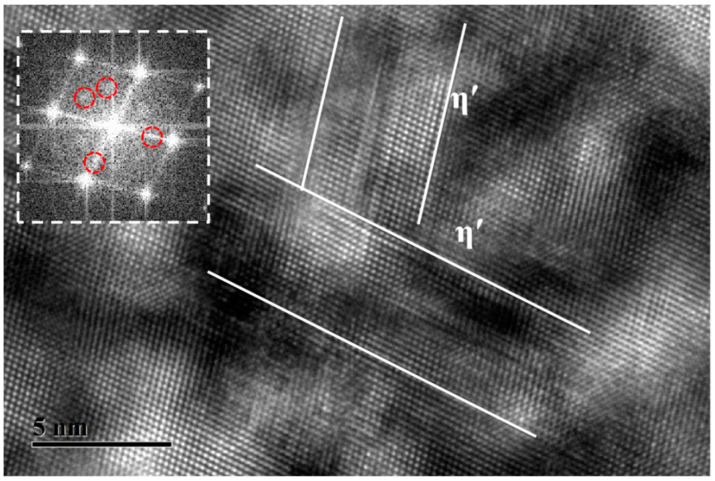
HRTEM image of the microstructure of the H-200-20 specimen (The red circles indicated the diffraction spots of the precipitated phase).

When heated to 200 °C, it can be inferred from the hardness level that the specimen has entered into an overaged state. The corresponding high-resolution image with an FFT insert of the coarse precipitate is shown in Figure 10. HRTEM does not show an incoherent relationship between the plate-like precipitate and Al, indicating that the main precipitates in the slightly overaged specimen are still semi-coherent η′ phases. However, the conductivity of the H-200-20 specimen has improved to 36.3% IACS, which is 1.3% higher than that of the H-190-20 state. This once again indicates a decrease in lattice distortion because of the coarsening of η′. As can be seen with HRTEM, the aspect ratio of η′ phase has significantly decreased, and the atomic layer thickness increases, resulting in a decrease in strength.

## 5. Conclusions

During the heat treatment process of a large cross-section of AlZnMgCu alloy components, non-isothermal temperature fields are inevitable. The utility of non-isothermal temperature fields for the short-term and efficient heat treatment of aluminum alloy components has been a research hotspot for engineering applications. The current study investigated the precipitation behavior of a spray-deposited AlZnMgCu alloy during heat aging, with a focus on analyzing the effects of the heating rate and termination temperature *Ts* on the hardness and conductivity. The mechanical properties and exfoliation corrosion performance of the spray-deposited AlZnMgCu alloy under single-stage peak aging (SA), double-stage overaging (DA), regression and re-aging (RRA), and the heat aging effect are compared. The main conclusions are as follows.

The results on the changes in hardness and electrical conductivity under different heating rates indicate that with increasing annealing temperature, the hardness of the spray-deposited AlZnMgCu alloy initially rises to a peak and then sharply decreases, while the electrical conductivity continues to increase. For heating rates of 10 °C/h, 20 °C/h, and 40 °C/h, the corresponding annealing temperatures are 190 °C, 200 °C, and 210 °C.

The findings regarding the mechanical properties and corrosion resistance of heat-aged samples under different heating rates demonstrate that a heat-aging process with a rate of 20 °C/h to reach 200 °C can yield optimal mechanical properties and corrosion resistance. This performance closely approaches that of RRA-treated samples, meeting aerospace application requirements for corrosion resistance.

In integrating microstructural observations, it is concluded that an optimal heat-aging process involves a rate of 20 °C/h to 200 °C and finally water cooling. The process can achieve an outstanding performance within just 8 h, effectively reducing the heating time from 40 h to 8 h while meeting the stringent requirements for aerospace applications. This establishes a highly efficient heat treatment process for spray-deposited AlZnMgCu alloy plates.

## Figures and Tables

**Table 1:** The comparison of comprehensive properties and time consumption of the spray-deposited AlZnMgCu alloy under different aging treatments.

Treatments	UTS (MPa)	YS (MPa)	Elongation (%)	Exfoliation Corrosion Degree	Time Consumption
SA	659.9	596	13.1	ED	16 h
DA	524.1	470	16.5	EA	30 h
RRA	684.0	643	13.3	EA	40 h
H190-10	619.1	600	10.5	EB	15 h
H200-20	642.3	616	11.3	EB	8 h
H210-40	627.0	602	11.1	EC	4.3 h

Noting: RRA—Retrogression and Re-aging, SA—single aging, DA—double aging, UTS—ultimate tensile strength, YS—yield strength.

**Table 2:** Typical parameters of matrix precipitates (MPts), grain boundary precipitates, and precipitate-free zones during heat-aging treatments.

Treatment	MPt Average Size/nm	GBP Spacing/nm	PFZ Width/nm	Hardness /HV	Electrical Conductivity/% IACS
H-160-20	3.9	-	-	184.1	30.3
H-190-20	7.2	10.8	-	190.4	34.9
H-200-20	8.5	13.7	25	187.4	36.3
H-210-20	10.9	22.6	27.2	167.7	39.4

## Data Availability

The original contributions presented in the study are included in the article, further inquiries can be directed to the corresponding authors.
